# Supplementary material for: Causal Relationship between Aging and Anorexia Nervosa: A White-Matter-Microstructure-Mediated Mendelian Randomization Analysis
Source: Biomedicines. 2024 Aug 16;12(8):1874. doi: 10.3390/biomedicines12081874 (PMC11351342; doi:10.3390/biomedicines12081874)
Supplement: Supplementary file 1 [file biomedicines-12-01874-s001.zip › Supplementary Figure/Figure S4/Singe SNP plot of MR analysis for TL to ubm-b-1735.pdf]

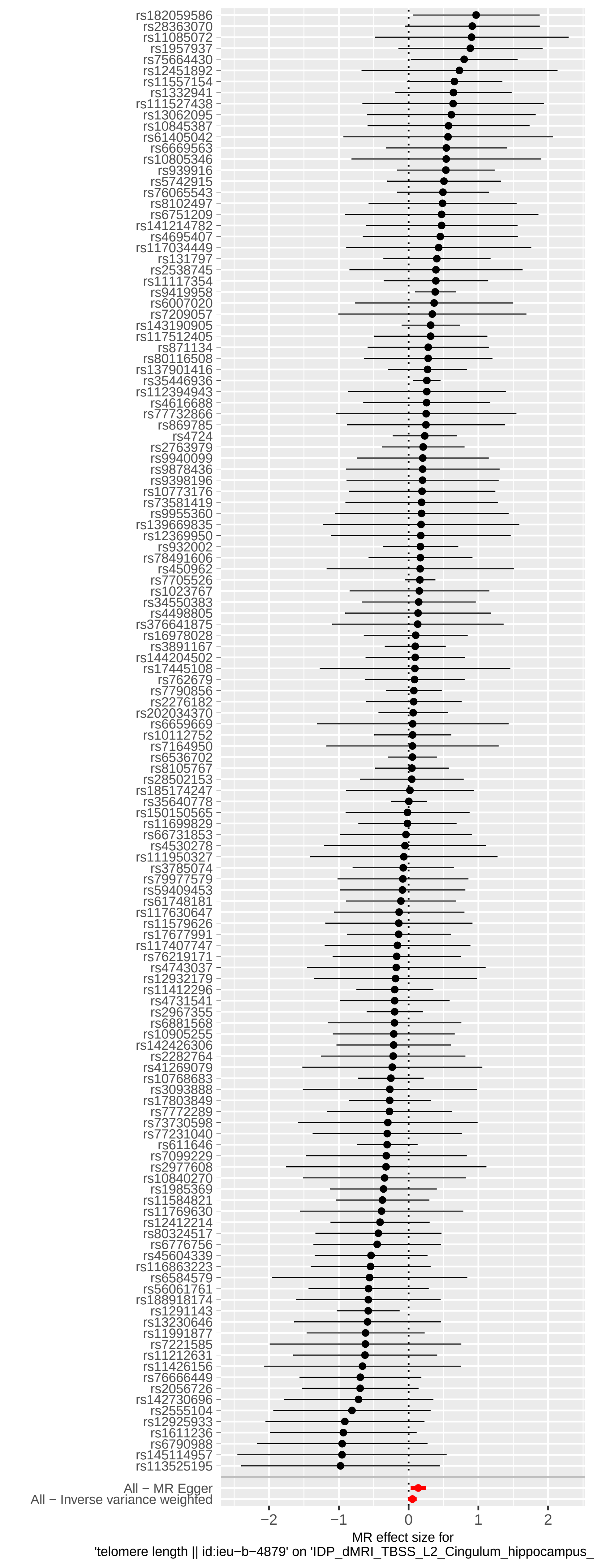
rs182059586
rs28363070
rs11085072
rs1957937
rs75664430
rs12451892
rs11557154
rs1332941
rs111527438
rs13062095
rs10845387
rs61405042
rs6669563
rs10805346
rs939916
rs5742915
rs76065543
rs8102497
rs6751209
rs141214782
rs4695407
rs117034449
rs131797
rs2538745
rs11117354
rs9419958
rs6007020
rs7209057
rs143190905
rs117512405
rs871134
rs80116508
rs137901416
rs35446936
rs112394943
rs4616688
rs77732866
rs869785
rs4724
rs2763979
rs9940099
rs9878436
rs9398196
rs10773176
rs73581419
rs9955360
rs139669835
rs12369950
rs932002
rs78491606
rs450962
rs7705526
rs1023767
rs34550383
rs4498805
rs376641875
rs16978028
rs3891167
rs144204502
rs17445108
rs762679
rs7790856
rs2276182
rs202034370
rs6659669
rs10112752
rs7164950
rs6536702
rs8105767
rs28502153
rs185174247
rs35640778
rs150150565
rs11699829
rs66731853
rs4530278
rs111950327
rs3785074
rs79977579
rs59409453
rs61748181
rs117630647
rs11579626
rs17677991
rs117407747
rs76219171
rs4743037
rs12932179
rs11412296
rs4731541
rs2967355
rs6881568
rs10905255
rs142426306
rs2282764
rs41269079
rs10768683
rs3093888
rs17803849
rs7772289
rs73730598
rs77231040
rs611646
rs7099229
rs2977608
rs10840270
rs1985369
rs11584821
rs11769630
rs12412214
rs80324517
rs6776756
rs45604339
rs116863223
rs6584579
rs56061761
rs188918174
rs1291143
rs13230646
rs11991877
rs7221585
rs11212631
rs11426156
rs76666449
rs2056726
rs142730696
rs2555104
rs12925933
rs1611236
rs6790988
rs145114957
rs113525195
All − MR Egger
All − Inverse variance weighted
−2
−1
0
1
2
MR effect size for
'telomere length || id:ieu−b−4879' on 'IDP_dMRI_TBSS_L2_Cingulum_hippocampus_
